# Chaos Time Series Prediction Based on Membrane Optimization Algorithms

**DOI:** 10.1155/2015/589093

**Published:** 2015-03-22

**Authors:** Meng Li, Liangzhong Yi, Zheng Pei, Zhisheng Gao, Hong Peng

**Affiliations:** ^1^School of Radio Management Technology Research Center, Xihua University, Chengdu 610039, China; ^2^School of Computer Science and Technology, Sichuan Police College, Luzhou 646000, China

## Abstract

This paper puts forward a prediction model based on membrane computing optimization algorithm for chaos time series; the model optimizes simultaneously the parameters of phase space reconstruction (*τ*, *m*) and least squares support vector machine (LS-SVM) (*γ*, *σ*) by using membrane computing optimization algorithm. It is an important basis for spectrum management to predict accurately the change trend of parameters in the electromagnetic environment, which can help decision makers to adopt an optimal action. Then, the model presented in this paper is used to forecast band occupancy rate of frequency modulation (FM) broadcasting band and interphone band. To show the applicability and superiority of the proposed model, this paper will compare the forecast model presented in it with conventional similar models. The experimental results show that whether single-step prediction or multistep prediction, the proposed model performs best based on three error measures, namely, normalized mean square error (NMSE), root mean square error (RMSE), and mean absolute percentage error (MAPE).

## 1. Introduction

Chaotic time series is a kind of nonlinear dynamic phenomenon between certainty and randomness, in which Lyapunov exponent is adopted to decide whether a time series is chaos or not; that is, the time series is chaotic if its Lyapunov exponent is greater than zero [1]. Because it can be widely applied in real life, such as in the network traffic, earthquake prediction, and weather forecasting [2–5], chaotic time series prediction has become a hot spot, and many interesting results have been provided by a lot of researchers in recent years [6, 7].

Initially, the traditional statistical fitting methods, such as autoregressive (AR), moving average (MA), and autoregressive moving average (ARMA) models, have been used in chaotic time series prediction. However, due to the inherent linearity assumptions, the above conventional mathematical tools are not well suited for dealing with ill-defined and uncertain systems. With the recent development in chaos theory, numerous nonlinear systems have been identified to be chaotic despite their random behaviors, in which the local model is an important method for chaotic time series; the method projected chaotic time series into a multidimensional phase space, which is then divided into several subspaces where the mapping function is approximated by means of local approximation [8–10]. Chaotic time series prediction based on nonlinear systems shows in general superior performance over the traditional statistical fitting methods. As another alternative in dealing with nonlinear systems, support vector machine (SVM) was proposed in [11, 12] based on the principles of the statistical VC (Vapnik Chervonenkis) dimensional theory and structural risk minimization. SVM can better solve problems such as nonlinear, dimension disaster, and good performance for the small sample. It will be widely used in face recognition, speech recognition [13–15], and so forth. Because of its universal approximation capabilities, recently, least squares support vector machine (LS-SVM) [16] is applied to predict chaotic time series [17, 18]. In the model, firstly, the phase space reconstruction technique of chaotic theory is used to reconstruct the nonlinear data; then the least squares support vector machine regression is applied in multidimensional phase space.

Formally, phase space reconstruction method is succeeded by delay time and embedding dimension; that is, for a given time series *x*_1_,…, *x*_*n*−1_, *x*_*n*_ (*n* is the number of the data), by using delay time and embedding dimension, the phase points after reconstruction of the time series are *X*_*i*_ = [*x*_*i*−(*m*−1)*τ*_,…, *x*_*i*−*τ*_, *x*_*i*_]  (*i* = 1,…, *M* − 1, *M*), where *τ* is delay time, *m* is embedding dimension, and *M* is the number of phase space points [19]. Accordingly, the prediction value of next time *t* + 1 based on LS-SVM can be expressed as

(1) $$\begin{array}{c} x_{t+1}=f\left(X_{t}\right), \end{array}$$

where *f*(·) is regression estimates function.

In applications, there are two key problems in the prediction model based on LS-SVM. One is the choice of delay time (*τ*) and embedding dimension (*m*) in the process of phase space reconstruction. Another is the selection of kernel function and its relevant parameters [20]. The phase space reconstruction is used to express out the trace of the evolution of chaotic time series without singular; namely, chaotic time series is projected into a multidimensional phase space. Kernel function is associated with learning and modeling for the data set of phase space reconstruction to forecast accurately the future value. A large number of studies have shown that the selection of delay time (*τ*) and embedding dimension (*m*) in phase space reconstruction has a direct impact on prediction results of chaotic time series [21]. If *τ* is too small in the delay neighbor element of the phase space, there will be information redundancy. If it is too big, *τ* leads to loss of information; the track of signals will occur folding phenomenon. Similarly, if *m* is too small, it is not enough to show the detailed structure of chaotic systems. If *m* is too big, the calculation will become complicated and cause the impact of noise.

**Figure 1:**
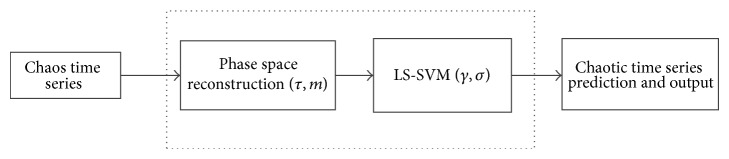
The flow chart of chaotic time series prediction.

LS-SVM learning performance is largely dependent on the choice of kernel function. A large number of studies have shown that, with the lack of a priori knowledge of specific issues, the overall performance of the radial basis kernel function model is better than other kernel function models and hence this paper selects the radial basis kernel function as the kernel function of LS-SVM. So in the model, there are two parameters (cost factor (*γ*) and kernel parameter (*σ*)) that need to be identified; cost factor *γ* is generally used to control the model complexity and compromise of approximation error, which is commonly in [1,1000]. Kernel parameter *σ* reflects the structure of high-dimensional feature space and affects the generalization ability of the system; when the value of *σ* is too small, it will occur over-learning phenomenon and poor generalization, while the value of *σ* is too large, it will emerge less learning phenomenon; the range of *σ* is in [0.1,10000] [22]. Currently, there are mainly two ideas for optimization of the parameters of the phase space reconstruction (*τ*, *m*) and LS-SVM (*γ*, *σ*). One is that the parameters were optimized separately as shown in Figure 1, in which, firstly, optimal delay time (*τ*) and embedding dimension (*m*) in the phase space are selected independently [19, 23–28] or at the same time [27, 29, 30]; then parameters *γ* and *σ* of the LS-SVM are selected by gradient descent method [31], genetic algorithm (GA) [32] or particle swarm optimization (PSO) [33], and so forth. Another idea is to optimize jointly the parameters, that is, the parameters (*τ*, *m*, *γ*, *σ*) as a whole to carry on the optimization [34].

Membrane systems presented in [35], also called *P* systems, are bioinspired computing models belonging to a broader family of so-called biological or natural computing [36, 37], which is a distributed and parallel computing model with hierarchy. Recently, membrane systems are widely used in many fields, such as in gasoline blending scheduling, radar emitter signals analyzing, and images skeletonizing [38–40]. This paper uses a membrane computing (cell-like membrane computing optimization algorithm) to optimize simultaneously the parameters of the phase space reconstruction and LS-SVM (namely, *τ*, *m*, *γ*, and *σ*). It is an important basis for spectrum management to predict accurately the change trend of parameters in the electromagnetic environment, which can help decision makers to develop an optimal action program. Then, using the model presented in this paper to predict band occupancy rate of frequency modulation (FM) broadcasting band and interphone band.

The rest of this paper is organized as follows.  Section 2 briefly reviews phase space reconstruction, LS-SVM regression, and membrane computing.  Section 3 introduces specifically the algorithm of parameters joint optimization about prediction model. In Section 4 the prediction model presented in this paper will be used to predict the parameters of electromagnetic environment. Conclusions are given in Section 5.

## 2. Preliminaries

### 2.1. Phase Space Reconstruction and LS-SVM Regression

Let the time series be {*x*_1_,…, *x*_*n*−1_, *x*_*n*_}; after the phase space reconstruction, the points in phase space can be expressed as [41, 42]

(2) $$\begin{array}{c} X_{i}=\left(x_{i},\ldots ,x_{i+(m-2)\tau },x_{i+(m-1)\tau }\right)\;\left(i=1,\ldots ,M-1,M\right), \end{array}$$

where *M* = *n* − (*m* − 1)*τ* is the number of phase space points, *τ* denotes the delay time, and *m* is embedding dimension.

Assume the given *l* samples data {(*X*_*i*_, *y*_*i*_)∣*i* = 1,…, *l* − 1, *l*}, where *X*_*i*_ ∈ *R* ^*d*^ is the sample input, *y*_*i*_ ∈ *R* is the sample output. The regression principle of LS-SVM can be explained as follows:

(3) $$\begin{array}{c} y\left(X\right)=\omega^{T}\Phi \left(X\right)+b, \end{array}$$

where Φ(·) is a nonlinear mapping from the input space to the feature space, *ω* is a vector of weight coefficients, and *b* is a bias constant.

The optimal hyperplane will be determined by the maximum geometry interval. Hence the LS-SVR problem can be transformed as follows [43]:

(4)  min⁡ J(ω,ζ)=12ωTω+12γ∑i=1lζi, s.t., yi=ωTΦ(Xi)+ζi+b, i=1,…,l−1,l,

where *ζ*_*i*_ are the error variables and *γ* is hyperparameter. The process of finding the optimal decision function is to determine the process parameters *ω* and *b*.

Introducing Lagrange multipliers, one can establish Lagrange functions as follows:

(5) $$\begin{array}{c} L=\frac{1}{2}\omega^{T}\omega +\frac{1}{2}\gamma \overset{l}{\underset{i=1}{\sum }}\zeta_{i}-\overset{l}{\underset{i=1}{\sum }}\alpha_{i}\left(\omega^{T}\Phi \left(X_{i}\right)+\zeta_{i}+b-y_{i}\right), \end{array}$$

where *α*_*i*_  (*i* = 1,…, *l* − 1, *l*) are the Lagrange multiplier. The conditions for optimality are given by

(6) $$\begin{array}{c} \frac{\partial L}{\partial \omega }=0,\;\;\frac{\partial L}{\partial b}=0,\;\;\frac{\partial L}{\partial \zeta_{i}}=0,\;\;\frac{\partial L}{\partial \alpha_{i}}=0. \end{array}$$

After elimination of the variables *ω* and *ζ*, a set of linear equations can be obtained:

(7) $$\begin{array}{c} \left(\begin{array}{cc} 0 & {I^{\prime }}^{T}\\ I^{\prime } & \Omega +\gamma^{-1}I \end{array}\right)\left(\begin{array}{c} b\\ \alpha \end{array}\right)=\left(\begin{array}{c} 0\\ Y \end{array}\right), \end{array}$$

where *I*′ = (1,…,1,1)^*T*^ ∈ *R* ^*l*^, *I* ∈ *R* ^*l*×*l*^ denotes a unit matrix, *α* = (*α*_1_,…,*α*_*l*−1_, *α*_*l*_)^*T*^, *Y* = (*y*_1_,…,*y*_*l*−1_, *y*_*l*_)^*T*^, and *Ω*_*i*,*j*_ = Φ(*X*_*i*_)^*T*^Φ(*X*_*j*_)  (*i*, *j* = 1,…, *l* − 1, *l*).

Then, LS-SVM regression model is expressed as

(8) $$\begin{array}{c} f\left(X\right)=\overset{l}{\underset{i=1}{\sum }}\alpha_{i}\Phi {\left(X_{i}\right)}^{T}\Phi \left(X_{j}\right)+b. \end{array}$$

The mapping function Φ(·) can be paraphrased by a kernel function *K*(·, ·) because of the application of* Mercer's* theorem, which means that *K*(·, ·)  (*i* = 1,…, *l* − 1, *l*) are any kernel functions satisfying the Mercer condition, and the Mercers condition has been applied:

(9) $$\begin{array}{c} K\left(X_{i},X_{j}\right)=\Phi {\left(X_{i}\right)}^{T}\Phi \left(X_{j}\right)\;\left(i,j=1,\ldots ,l-1,l\right). \end{array}$$

This finally results in the following LS-SVM model for function regression:

(10) $$\begin{array}{c} f\left(X\right)=\overset{n}{\underset{i=1}{\sum }}\alpha_{i}K\left(X,X_{i}\right)+b. \end{array}$$

As shown in Figure 1, the prediction model of phase space reconstruction and LS-SVM regression mainly has two steps. First, select the delay time (*τ*), embedding dimension (*m*), and LS-SVM parameters (*γ* and *σ*). The phase space reconstruction technique is used to determine the training sample pairs based on the parameters *τ* and *m* which are determined. Assuming the time series is {*x*_1_, *x*_2_,…, *x*_*N*+1_}, the training sample set of attributes is as follows:

(11) $$\begin{array}{c} R=\left(\begin{array}{cccc} x_{1} & x_{1+\tau } & \cdots & x_{1+(m-1)\tau }\\ x_{2} & x_{2+\tau } & \cdots & x_{2+(m-1)\tau }\\ \vdots & \vdots & \vdots & \vdots \\ x_{N-(m-1)\tau } & x_{N-(m-1)\tau +\tau } & \cdots & x_{N-(m-1)\tau +(m-1)\tau } \end{array}\right). \end{array}$$

The training sample set of labels is *A* = (*x*_1+(*m* − 1)*τ*+1_, *x*_2+(*m* − 1)*τ*+1_,…,*x*_*N*+1_)^*T*^. Second, predict future point *x*_*i*_ in the future. Select the attribute sample of the previous time as input in the phase space and use the trained LS-SVM model to obtain the predicted value of the moment.

### 2.2. Membrane Computing

Membrane computing (namely, *p* systems) arises as a new model of computation, inspired by the way that cells are structured into vesicles and abstracting the chemical reactions taking place inside them [44]. It is a branch of molecular computing that aims to develop models and paradigms that are biologically motivated. There has been a flurry of research activities in this area in recent years [45]. Because of the built-in nature of maximal parallelism inherent on the models, *p* systems have a great potential for implementing massively concurrent systems in an efficient way that would allow us to solve currently intractable problems.

A membrane system with degree *d*  (*d* > 0) can be expressed as

(12) $$\begin{array}{c} \prod =\left(V,T,C,\mu ,W_{1},\ldots ,W_{d},\left(R_{1},\rho_{1}\right),\ldots ,\left(R_{d},\rho_{d}\right)\right), \end{array}$$

where *V* is an alphabet, whose elements are called objects, *T* denotes the output alphabet, *C* is a catalyst, which does not exhibit any change in the course of evolution, but some reaction must have its participation, *μ* is the membrane structure, which can be shown by [], *W*_*i*_ denotes multiple sets of objects in the membrane structure, and (*R*_*i*_, *ρ*_*i*_) are the set of rules, in which *R*_*i*_ and *ρ*_*i*_ denote rule and the priority of the rule, respectively.

**Figure 2:**
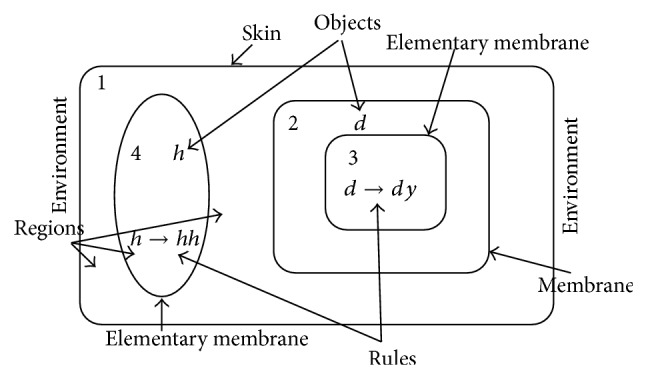
Simple membrane structure diagram.

In general, *p* system contains three core elements: membrane structure, object multiple sets, and evolution rules. A membrane system with given membrane structure, evolution rules, and decided objects will be performed in the form of nondeterministic and maximum parallel for the evolution rules. When all the objects are exhausted, the rules are no longer executed, the system downtime. A typical membrane system consists of cell-like membranes placed inside a unique “skin” membrane. Multisets of objects—usually strings of symbols—and a set of evolution rules are placed inside the regions delimited by the membranes. Each object can be transformed into other objects, can pass through a membrane, or can dissolve or create membranes. The evolution between system configurations is done nondeterministically by applying the rules in parallel for all objects able to evolve [46]. As shown in Figure 2, a simple membrane structure diagram can be shown by [[[]_3_]_2_[]_4_]_1_. The skin membrane, which is the outermost membrane of this structure, separates the system from its environment. Several membranes, each of which defines a region, are placed inside the skin membrane. Elementary membranes do not contain any membrane. Each region forms a different compartment of the membrane structure and contains a multiset of objects or membranes. Where *h* and *d* denote objects, *h* → *hh* and *d* → *dy* are rules.

## 3. Parameters Joint Optimization Algorithm Based on Membrane Computing

**Figure 3:**
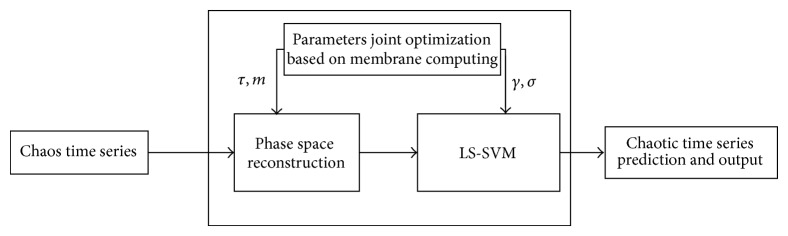
The structure of P-LSSVM prediction model.

The optimization algorithm based on cell-like membrane computing is an important branch of membrane computing. It is an intelligent optimization algorithm inspired by the mechanism and the function of biological cells and based on the existing framework of membrane computing. The steps generally are membrane structure establishment, the objects generation and evolution, and so forth. Shown in Figure 3 is the structure of P-LSSVM prediction model, with the initial objects as initial parameters of prediction model; these parameters are substituted into the phase space reconstruction and LS-SVM model. Then, parameters joint optimization algorithm based on membrane computing is used to decide the best combination of parameters. Algorithm specific process is as shown in Figure 3.

### 3.1. The Establishment of the Cellular Membrane Structure and the Generation of Objects

**Figure 4:**
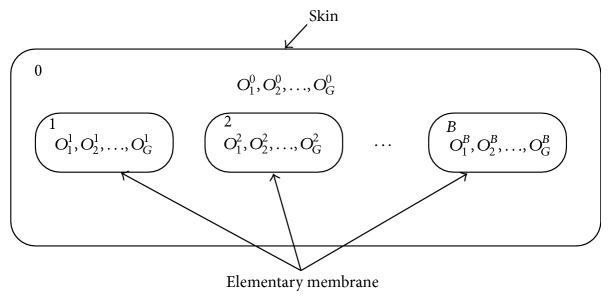
Membrane structure.

As shown in Figure 4, this paper adopts two layers structure for membrane, a skin contains *B* basic membrane, generate initial objects in each membrane. Generally, *p* system uses character or character string to encode, real number encoding are adopted in here, which can reduce the trouble of decode. For instance, *O* = (*o*_1_, *o*_2_, *o*_3_, *o*_4_), where *O* is an object and *o*_1_, *o*_2_, *o*_3_, and *o*_4_ denote *τ*, *m*, *γ*, and *σ*, respectively. We see each object as a solution of the optimization problem. Evolution of each membrane according to its own rules, all the membrane are executed in parallel. The final optimal results are output through the skin, that is, the optimal solution.

### 3.2. Construct the Fitness Function

The goal of cell-like membrane computing optimization algorithm is to find the most suitable combination of parameters (*τ*, *m*, *γ*, and *σ*) in order to establish the optimal forecasting model. In this paper, we used the root mean square prediction error (RMSE) to construct the fitness function. That is, *f* = 1/RMSE, $\text{RMSE}=\sqrt{(1/Z)\sum_{i=1}^{Z}{\left(y_{i}-{\hat{y}}_{i}\right)}^{2}}$, where *Z* denotes the number of prediction points and *y*_*i*_, ${\hat{y}}_{i}$ represent the real values and predicted values, respectively.

### 3.3. Operation Rules

The basic rules of cellular membrane computing optimization method are selection, crossover, mutation, and communication [47]. The specific form is as follows.

(1) Selection rule: the rule of selection copies the objects to the next generation according to the size of the string. The size of the string is not the three-dimensional size of particles in biological cells but the value of the fitness function. Here, wheel disk method is used to select objects to the next generation.

(2) Crossover rule: for any two objects *O*_*i*_ = (*o*_*i*1_, *o*_*i*2_, *o*_*i*3_, *o*_*i*4_) and *O*_*j*_ = (*o*_*j*1_, *o*_*j*2_, *o*_*j*3_, *o*_*j*4_), use cross rule to obtain new object *O*_*k*_ = (*o*_*k*1_, *o*_*k*2_, *o*_*k*3_, *o*_*k*4_):

(13) ok1=r×oi1+(1−r)×oj1,ok2=r×oi2+(1−r)×oj2,ok3=r×oi3+(1−r)×oj3,ok4=r×oi4+(1−r)×oj4,

where *r* is a random number in (0,1).

(3) Mutation rule: in evolution, according to a certain mutation probability, replace the worst *t* objects with randomly generated *t* objects. Mutating rule is described as follows:

(14) Ri,mutation:qmin⁡1,qmin⁡2,…,qmin⁡ti⟶qinit1,qinit2,…,qinitti,

where []_*i*_ denotes membrane *i*, *q*_min⁡1_, *q*_min⁡2_,…, *q*_min⁡*t*_ are *t* objects where fitness is the smallest in membrane *i*, and *q*_init1_, *q*_init2_,…, *q*_init*t*_ are randomly generated *t* objects.

(4) Communication rule: each membrane *i* will transport the best *L* objects out of the membrane, while the best *L* objects of foreign membrane are brought into the membrane *i* [48]. This rule can be expressed as follows:

(15) Ri,communication=Ri,communication1∪Ri,communication2,Ri,communication1:qmax⁡1,qmax⁡2,…,qmax⁡Li⟶iqmax⁡1,qmax⁡2,…,qmax⁡L,Ri,communication2:iqmax⁡1′,qmax⁡2′,…,qmax⁡L′⟶qmax⁡1′,qmax⁡2′,…,qmax⁡L′i,

where []_*i*_ denotes membrane *i*, *q*_max⁡1_, *q*_max⁡2_,…, *q*_max⁡*L*_ are the best *L* objects in membrane *i*, and *q*_max⁡1_′, *q*_max⁡2_′,…, *q*_max⁡*L*_′ are the best *L* objects out of membrane *i*.

### 3.4. Parameters Joint Optimization Algorithm Specific Steps in P-LSSVM Model

First of all, generate the initial objects as initial parameters of prediction model; then apply evolutionary rules to evolve until the stop conditions are met; all membranes are operating in parallel. Finally, output the fitness of the best object by the skin membrane, that is, the optimal solution. Specifically, consider the following.

**Step 1 .**

Initialize parameters and build cellular membrane structure.

(1) Initialization: the number of elementary membranes is *B*, the number of objects in each membrane is *G*, the largest number of iterations is *Max*⁡*T*, crossover probability is *P*_*c*_, mutation probability is *P*_*m*_, and the current iteration number is *k*, and so forth.

(2) Create membrane structure as shown in Figure 4, generating randomly *G* objects in each membrane; each object represents a set of parameters' combination, expressed in decimal coding.

**Step 2 .**

Optimize each membrane in turn.

(1) Every object in the membrane as a set of parameters (*τ*, *m*, *γ*, and *σ*) of P-LSSVM model; calculate the fitness of each object by training data and save the optimal object and its fitness.

(2) Use the reproduction, crossover, and mutation rules to evolve.

**Step 3 .**

Make use of communication rules; each membrane will transport the best *L* objects out of the membrane; at the same time, the best *L* objects outside the membrane will be shipped into the membrane.

**Step 4 .**

Determine whether the termination condition is satisfied, that is, whether it reaches the maximum number of iterations, when the number of iterations is less than the maximum number of iterations to continue iteration or stop iteration.

**Step 5 .**

The optimal object is output from the skin membrane.

## 4. Electromagnetic Environment Parameters Predictions Based on P-LSSVM Model

Electromagnetic spectrum is a fundamental strategic resource to support the national economy and national defense construction, along with the rapid development of information technology and it is widely used in various fields such as economic development, national defense construction, and social life [49]. Strategic value and basic role increasingly highlight in the electromagnetic spectrum, with frequency contradictions increasingly prominent between countries, departments, and military and space businesses [50]. It is an important basis for spectrum management to control comprehensively the change trend of parameters in the electromagnetic environment of country or region [51]. It is the basis to master the frequency information for the frequency planning, frequency allocation, and sharing service frequency recovery work. The situation of electromagnetic environment can be reflected by the electromagnetic environment indicator parameters; these parameters mainly include band occupancy rate, channel occupancy rate, large-signal ratio, frequency offset, and the field strength. A large number of experiment shown that time series data with chaotic in the electromagnetic environment. Hence, we used the proposed prediction model to predict the indicator parameters of the electromagnetic environment. The experimental results show that the prediction model proposed in this paper is reasonable and effective.

Here, we chose the band occupancy rate to do the test. Band occupancy rate is calculated as follows: extracting all the signal points in the spectrum data, the signals point are merged with distance less than bandwidth by the below formula to calculate the band occupancy rate (Occupy_Freband_):

(16) $$\begin{array}{c} {\text{Occupy}}_{\text{Freband}}=\frac{S_{n}*F_{w}}{F_{\text{end}}-F_{\text{begin}}}, \end{array}$$

where *S*_*n*_ denotes the total number of signals judged, *F*_*w*_ is necessary bandwidth in this band for the type of specified business, *F*_begin_ is the start frequency point, and *F*_end_ is the cutoff frequency point.

### 4.1. Experimental Data Sources

**Figure 5:**
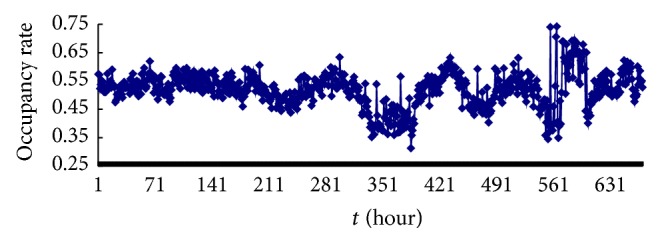
Band occupancy rate data of FM radio band.

**Figure 6:**
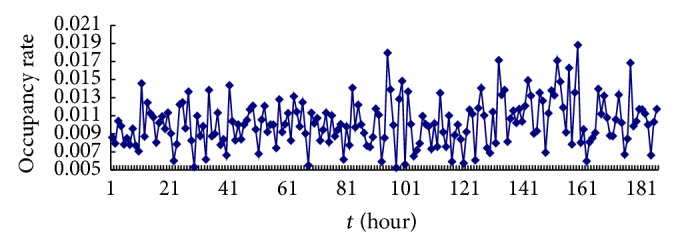
Band occupancy rate data of interphone band.

In this paper, we adopt digital receiver EM100 which was provided by German Rohde & Schwarz Company and fixed radio monitoring station of Xihua University to collect data for the experiment. We collected data including frequency modulation (FM) broadcasting band and interphone band. As shown in Figures 5 and 6, in which the vertical axis denotes band occupancy rate, the horizontal axis represents the collection time, and left picture shows the data of band occupancy rate in FM broadcasting band, we collected for 680 hours, that is, obtaining 680 pieces of data. Right figure indicates acquisition data of band occupancy rate in interphone band; we continuously collected for 187 hours, that is, gaining 187 pieces of data. In order to facilitate narration, here we put the band data of FM broadcasting band and interphone band, denoted by “data set 1” and “data set 2,” respectively. Use the method of small amount of data to calculate the maximum Lyapunov index of two groups of data which are *λ*_1_ = 0.126 and *λ*_2_ = 0.14, respectively, which show the time series with chaos.

### 4.2. Data Preprocessing

This paper mainly uses the Grubbs criteria to deal with the abnormal data; the method is as follows: let *q*(*h*, *d*) be the sequence of the collected data, with the time interval between two data collections *t* = 1 hour, where *h* = 0,…, 22,23 denote 24 hours of a day, *d* = 1,…, *H* − 1, *H* represents date code in total days of data collection *H*, and *q* denotes the collected data. Using data set denoted by *Q* = *q*_1_, *q*_2_,…, *q*_*t*_,…, for each time point *h*, we can get the expectation and variance of data sequence *q*(*h*, *d*); the formula is as follows:

(17) $$\begin{array}{c} E\left(h\right)=\frac{1}{S}\overset{S}{\underset{k=1}{\sum }}q\left(h,d\right),\\ D\left(h\right)=\sigma_{i}^{2}=\frac{1}{S}\overset{S}{\underset{k=1}{\sum }}{\left[q\left(h,d\right)-E\left(h\right)\right]}^{2}, \end{array}$$

where *S* denotes the length of a unit.

According to the above two formulas, combined with Grubbs criteria, if the sample point meet to

(18) $$\begin{array}{c} \left|q\left(h,d\right)-E\left(h\right)\right|\geq G\left(n,\varepsilon \right)\sigma_{i}. \end{array}$$

The sample point should be removed, where *G*(*n*, *ε*) is the critical value of Grubbs criteria; it can be obtained by looking at Grubbs table; *ε* denotes the significance level; usually significance level *ε* = 0.05.

The Grubbs criteria are used to deal with “data set 1” and “data set 2,” respectively. For the “data set 1” after processing with Grubbs criteria, the remaining 653 pieces of data, we use the front 600 pieces of data as the training data, determining the best parameters combination, and the surplus 53 pieces of data as test data, testing the prediction accuracy of the model. For the “data set 2” after processing with Grubbs criteria, the remaining 180 pieces of data, we use the front 150 pieces of data as the training data, determining the best parameters combination, and the surplus 30 pieces of data as test data, testing the prediction accuracy of the model.

### 4.3. Reference Model and Evaluation Criteria

In order to verify the validity of the model, this paper will compare the prediction model (P-LSSVM) proposed in this paper with conventional similar prediction model. The first reference model is the parameters joint optimization based on genetic algorithm for chaos time series prediction (GA-LSSVM) [34]. The second reference model uses the mutual information method and Cao method to get the best delay time *τ* and embedding dimension *m*, respectively. And then use grid search method to obtain LS-SVM parameters (*γ* and *σ*) (denoted as M-C-LSSVM) [19]. The third reference model uses the mutual information method and false nearest neighbor method to calculate the optimal delay time *τ* and embedding dimension *m*, respectively. And then, use genetic algorithm to get the optimal combination parameters of LS-SVM (*γ* and *σ*) (denoted as M-F-LSSVM) [27]. The fourth reference model uses C-C method to seek simultaneously the best delay time *τ* and embedding dimension *m*. Then the optimal parameters of LS-SVM (*γ* and *σ*) by using genetic algorithm (denoted as C-C-LSSVM) [27, 52].

Meanwhile, this paper uses three evaluation criteria: normalized mean square error (NMSE), root mean square error (RMSE), and mean absolute percentage error (MAPE). NMSE, RMSE, and MAPE are defined, respectively, as follows:

(19) NMSE=1σ2Z∑i=1Zyi−y^i2, σ2=1Z−1∑i=1Zyi−y−2,y−=∑i=1Zyi,RMSE=1Z∑i=1Zyi−y^i2,MAPE=1Z∑i=1Zyi−y^iyi×100%,

where *Z* is the number of prediction points, $\overset{-}{y}$ is the average value, and *y*_*i*_ and ${\hat{y}}_{i}$ denote the real value and the predicted value of *i*th point, respectively.

### 4.4. Experimental Results

In this paper, the scope of parameters *τ*, *m*, *γ*, and *σ* is [1,8], [3,17], [1,1000], and [0.1,10000], respectively. In the process of evolution, the other parameters are set as follows: the number of elementary membranes *B* = 20, the number of objects in each membrane *G* = 100, evolution algebra *Max*⁡*T* = 1000, crossover probability *P*_*c*_ = 0.85, and mutation probability *P*_*m*_ = 0.05. The optimal parameters combinations of each model are shown in Tables 1 and 2.

#### 4.4.1. Single-Step Prediction

**Figure 7:**
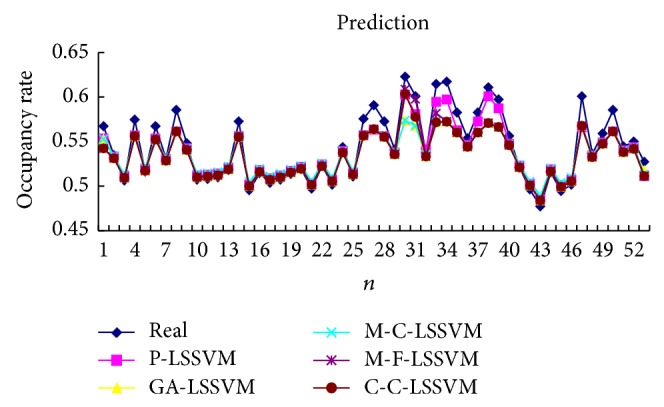
Five models predicted diagram for FM broadcasting band.

**Figure 8:**
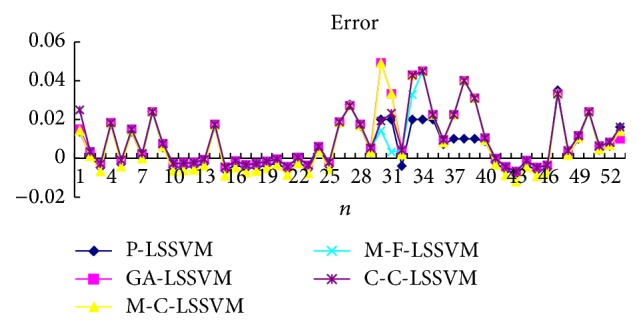
Five models predicted error diagram for FM broadcasting band.

**Figure 9:**
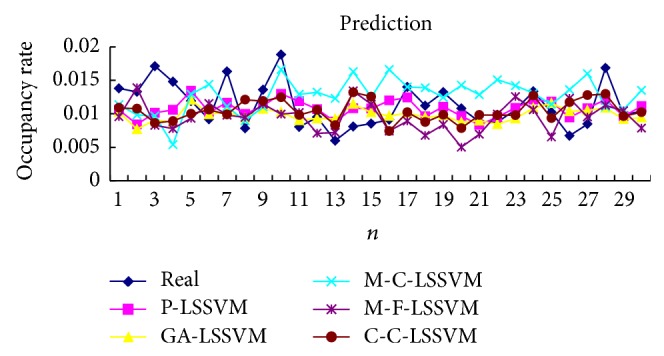
Five models predicted diagram for interphone band.

**Figure 10:**
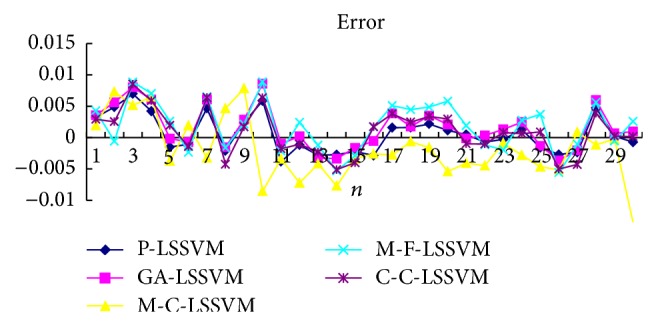
Five models predicted error diagram for interphone band.

Selecting the first point as input to obtain first predicted value, then the real value of the first point is added to the historical data, predicting the next point. And so, obtain the predicted value of all points. Prediction results of five models are shown in Tables 3, 4, 5, 6, 7, and 8 and Figures 7, 8, 9, and 10.

#### 4.4.2. Multistep Forecast

**Figure 11:**
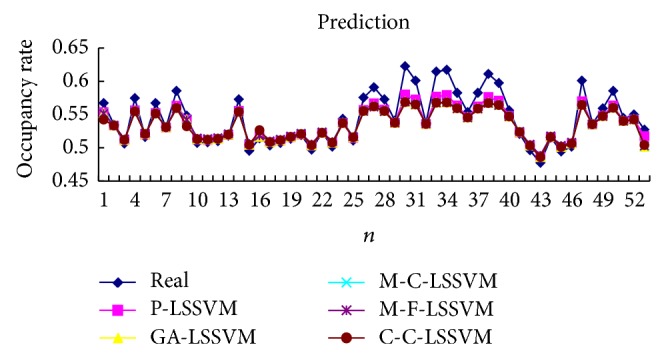
Five models predicted diagram for FM broadcasting band.

**Figure 12:**
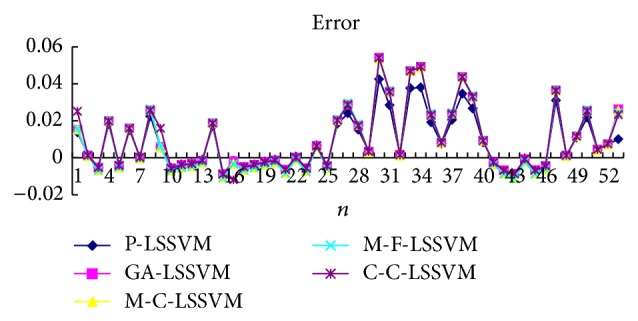
Five models predicted error diagram for FM broadcasting band.

**Figure 13:**
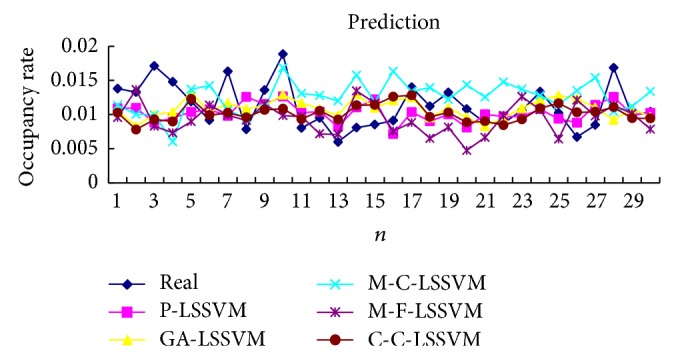
Five models predicted diagram for interphone band.

**Figure 14:**
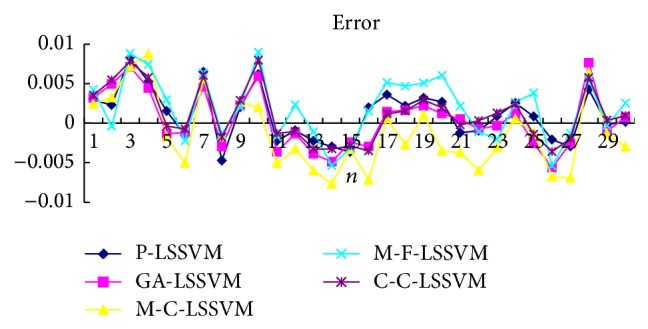
Five models predicted error diagram for interphone band.

Selecting a point as input to obtain predicted value, then the prediction value of the first point is added to the historical data, predicting next point. And so, obtain the predicted value of all points. Predicted results of five models are shown in Tables 9, 10, 11, 12, 13, and 14 and Figures 11, 12, 13, and 14.

### 4.5. Analysis of Experimental Results

The optimal parameters combinations of five models for FM broadcasting band and interphone band are shown in Tables 1 and 2, respectively. As seen from experimental results, we can find that the parameters *τ*, *m*, *γ*, and *σ* are very sensitive to prediction accuracy; the optimal parameters combination is P-LSSVM model; FM broadcasting bands are 7, 14, 163.1, and 6647.5. Interphone bands are 3, 8, 170.9, and 2162.1. It can be seen from predicted results diagram (Figures 7 to 14) that whether single-step prediction or multistep prediction five models get very good results. However the P-LSSVM model predicts curve best fit to real data and other curves relative deviation from far away. For five prediction models, respectively, run 10 times, computing the maximum, minimum, mean, and variance of error. As can be seen from predicted results in Tables 3 to 14, three kinds of models evaluation standard are RMSE, NMSE, and MAPE; the model proposed in this paper is the minimum. This shows that not only is the P-LSSVM model reasonable and correct, but prediction accuracy is also enhanced.

Comparing single-step prediction with multistep prediction, it can be found that the error of multistep prediction is larger than the single-step prediction, indicating that the effect of single-step prediction is better than multistep prediction. The reason is that errors exist in every step, and the accumulation of error will lead to decline in the overall prediction accuracy.

## 5. Conclusion

Modeling and prediction of chaotic time series has become a hot spot in the research field of the chaotic signal processing. In this paper, two defects were taken into consideration in the prediction model of LS-SVM for chaos time series prediction: on the one hand, ignoring the overall correlation of the parameters in prediction model and, on the other hand, considering the contact between the parameters, but the optimization methods have some limitations. For example, use genetic algorithm to solve the optimal parameter of prediction model, which itself has some limitations, such as falling into local optimum and iterative process complication. This paper puts forward a prediction model based on membrane computing optimization algorithm for chaos time series prediction; the model optimizes the parameters of phase space reconstruction and LS-SVM by using membrane computing optimization algorithm. Then, we used the model to forecast band occupancy rate of FM broadcasting band and interphone band. To show the applicability and superiority of the proposed model, this paper will compare the forecast model proposed in it with the traditional similar forecast model. The experimental results show that whether single-step prediction or multistep prediction, the proposed model performs best based on three error measures, namely, normalized mean square error (NMSE), root mean square error (RMSE), and mean absolute percentage error (MAPE). For deficiency in multistep prediction, in the next stage, we will further improve the prediction model or study other prediction models to improve the multistep prediction.

## Figures and Tables

**Table 1:** The optimal parameters combination of five models for FM broadcasting band.

Model	*τ*	*σ*	*γ*	*σ*
P-LSSVM	7	14	163.1	6647.5
GA-LSSVM	7	15	208.0	8250.4
M-C-LSSVM	3	15	744.1	6129.4
M-F-LSSVM	3	16	650.4	841.5
C-C-LSSVM	4	15	451.2	8082.0

**Table 2:** The optimal parameters combination of five models for interphone band.

Model	*τ*	*σ*	*γ*	*σ*
P-LSSVM	3	8	170.9	2162.1
GA-LSSVM	3	13	143.2	6532.1
M-C-LSSVM	2	14	614.3	1819.0
M-F-LSSVM	4	15	837.4	7497.8
C-C-LSSVM	2	12	798.8	6784.3

**Table 3:** Five models predicted error based on RMSE for FM broadcasting band.

Model	M-C-LSSVM	M-F-LSSVM	C-C-LSSVM	GA-LSSVM	P-LSSVM
run = 1	0.051	0.0486	0.0421	0.036	0.0217
run = 2	0.0501	0.0546	0.0464	0.0356	0.0252
run = 3	0.0489	0.0473	0.0425	0.0406	0.0261
run = 4	0.05	0.0545	0.0458	0.0347	0.0231
run = 5	0.0501	0.0588	0.0422	0.0407	0.023
run = 6	0.05	0.0583	0.0422	0.0365	0.0235
run = 7	0.0502	0.053	0.0439	0.0367	0.026
run = 8	0.0499	0.0545	0.0425	0.0379	0.028
run = 9	0.0495	0.0555	0.0423	0.0388	0.0282
run = 10	0.0484	0.0448	0.0474	0.0383	0.0281
Max.	0.051	0.0588	0.0474	0.0407	0.0282
Min.	0.0484	0.0448	0.0421	0.0347	0.0217
Ave.	0.0498	0.053	0.0437	0.0376	0.0253
Var.	5.00*E* − 07	2.00*E* − 05	4.00*E* − 06	4.00*E* − 06	6.00*E* − 06

**Table 4:** Five models predicted error based on NMSE for FM broadcasting band.

Model	M-C-LSSVM	M-F-LSSVM	C-C-LSSVM	GA-LSSVM	P-LSSVM
run = 1	1.6941	1.5377	1.5652	1.3773	0.6544
run = 2	1.6302	1.9412	1.4676	1.3536	0.8085
run = 3	1.558	1.9554	1.5819	1.3795	0.8467
run = 4	1.6263	1.9338	1.4227	1.2984	0.7149
run = 5	1.6344	2.2537	1.4727	1.0777	0.7078
run = 6	1.6274	2.2121	1.4762	1.4095	0.7245
run = 7	1.6377	1.8296	1.4892	1.4172	0.8432
run = 8	1.6191	1.9315	1.5916	1.4934	0.9421
run = 9	1.5819	2.0028	1.4784	0.9811	0.9517
run = 10	1.5246	1.8079	1.4673	1.2086	0.9465
Max.	1.6941	2.2537	1.5819	1.4934	0.9517
Min.	1.5246	1.5377	1.4227	0.9811	0.6544
Ave.	1.6144	1.9046	1.5023	1.2996	0.814
Var.	0.0022	0.0409	0.0034	0.0264	0.0122

**Table 5:** Five models predicted error based on MAPE for FM broadcasting band.

Model	M-C-LSSVM	M-F-LSSVM	C-C-LSSVM	GA-LSSVM	P-LSSVM
run = 1	0.0013	0.0015	0.001	9.58*E* − 04	8.19*E* − 04
run = 2	0.0013	0.0014	0.0013	9.05*E* − 04	4.94*E* − 004
run = 3	0.0015	0.0014	0.0013	9.79*E* − 04	4.57*E* − 04
run = 4	0.0013	0.0014	0.0014	8.39*E* − 04	4.87*E* − 04
run = 5	0.0015	0.0012	0.0012	8.08*E* − 04	8.54*E* − 04
run = 6	0.0015	0.0013	0.0013	8.92*E* − 04	6.12*E* − 04
run = 7	0.0015	0.0014	0.0013	8.78*E* − 04	7.84*E* − 04
run = 8	0.0013	0.0014	0.0013	9.00*E* − 04	5.94*E* − 04
run = 9	0.0015	0.0013	0.0012	8.30*E* − 04	7.01*E* − 04
run = 10	0.0015	0.0013	0.0012	8.40*E* − 04	6.32*E* − 04
Max.	0.0015	0.0015	0.0014	9.79*E* − 04	8.54*E* − 04
Min.	0.0013	0.0012	0.001	8.08*E* − 04	4.57*E* − 04
Ave.	0.0014	0.0014	0.0013	8.9*E* − 04	6.7*E* − 04
Var.	1.00*E* − 08	7.00*E* − 04	1.00*E* − 08	3.00*E* − 09	2.00*E* − 08

**Table 6:** Five models predicted error based on RMSE for interphone band.

Model	M-C-LSSVM	M-F-LSSVM	C-C-LSSVM	GA-LSSVM	P-LSSVM
run = 1	0.0041	0.0044	0.0038	0.003	0.0023
run = 2	0.0042	0.0045	0.0037	0.0026	0.0023
run = 3	0.0039	0.0044	0.0038	0.0026	0.0024
run = 4	0.004	0.004	0.0036	0.0026	0.0024
run = 5	0.0042	0.0044	0.0038	0.0027	0.0023
run = 6	0.004	0.0041	0.0037	0.0026	0.0025
run = 7	0.004	0.0044	0.0037	0.0026	0.0025
run = 8	0.004	0.0045	0.0037	0.0031	0.0026
run = 9	0.0041	0.004	0.0038	0.0025	0.0025
run = 10	0.0037	0.0045	0.0037	0.0026	0.0024
Max.	0.0042	0.0045	0.0038	0.0031	0.0026
Min.	0.0037	0.004	0.0036	0.0025	0.0023
Ave.	0.004	0.0043	0.0037	0.0027	0.0024
Var.	2.00*E* − 08	4.00*E* − 08	5.00*E* − 09	4.00*E* − 08	1.00*E* − 08

**Table 7:** Five models predicted error based on NMSE for interphone band.

Model	M-C-LSSVM	M-F-LSSVM	C-C-LSSVM	GA-LSSVM	P-LSSVM
run = 1	1.2458	1.7948	1.3449	1.0367	1.1577
run = 2	1.5297	1.8191	1.2826	1.0811	0.9321
run = 3	1.6352	1.7841	1.3119	1.0656	0.9817
run = 4	1.3912	1.4753	1.1894	1.0988	0.986
run = 5	1.4478	1.777	1.3191	1.2575	1.0121
run = 6	1.6357	1.5738	1.2857	1.2072	1.0362
run = 7	1.4753	1.7815	1.2601	1.0642	1.0053
run = 8	1.4478	1.8229	1.2627	1.0729	0.9984
run = 9	1.4534	1.4834	1.3027	1.079	1.0025
run = 10	1.5245	1.8605	1.412	1.023	0.975
Max.	1.6357	1.8605	1.4092	1.367	1.1577
Min.	1.2458	1.4753	1.1894	1.023	0.9321
Ave.	1.4786	1.7173	1.2971	1.0986	1.0087
Var.	0.0131	0.0216	0.0034	0.0056	0.0035

**Table 8:** Five models predicted error based on MASE for interphone band.

Model	M-C-LSSVM	M-F-LSSVM	C-C-LSSVM	GA-LSSVM	P-LSSVM
run = 1	0.0094	0.0074	0.0044	0.0024	0.0017
run = 2	0.0093	0.0074	0.0038	0.0025	0.0015
run = 3	0.0078	0.0058	0.0032	0.0021	0.0015
run = 4	0.0086	0.0051	0.0048	0.0032	0.0011
run = 5	0.0077	0.0067	0.0029	0.0031	0.0014
run = 6	0.0045	0.0046	0.0041	0.0026	0.0014
run = 7	0.0046	0.006	0.003	0.0023	0.0015
run = 8	0.0085	0.0065	0.0032	0.0024	0.0014
run = 9	0.0084	0.0049	0.0042	0.0033	0.0016
run = 10	0.0071	0.0073	0.0043	0.0023	0.0021
Max.	0.0094	0.0074	0.0048	0.0033	0.0021
Min.	0.0045	0.0046	0.0029	0.0021	0.0011
Ave.	0.0076	0.0062	0.0038	0.0026	0.0015
Var.	3.00*E* − 06	1.00*E* − 06	4.00*E* − 07	2.00*E* − 07	7.00*E* − 08

**Table 9:** Five models predicted error based on RMSE for FM broadcasting band.

Model	M-C-LSSVM	M-F-LSSVM	C-C-LSSVM	GA-LSSVM	P-LSSVM
run = 1	0.1996	0.1833	0.1498	0.1576	0.1162
run = 2	0.1957	0.192	0.1528	0.1311	0.1055
run = 3	0.1759	0.1776	0.1635	0.1426	0.0824
run = 4	0.1755	0.1617	0.1416	0.1366	0.1024
run = 5	0.1846	0.1756	0.1644	0.1397	0.1025
run = 6	0.1764	0.1534	0.1542	1.1458	0.0883
run = 7	0.1558	0.177	0.141	0.1468	0.1052
run = 8	0.1741	0.1716	0.154	0.1351	0.1076
run = 9	0.1777	0.1743	0.1639	0.1566	0.1066
run = 10	0.1768	0.1998	0.1641	0.1491	0.1065
Max.	0.1996	0.1998	0.1644	0.1576	0.1162
Min.	0.1558	0.1534	0.141	0.1311	0.0824
Ave.	0.1792	0.1766	0.1549	0.1441	0.1023
Var.	0.0001	0.0002	8.00*E* − 05	8.00*E* − 05	1.00*E* − 04

**Table 10:** Five models predicted error based on NMSE for FM broadcasting band.

Model	M-C-LSSVM	M-F-LSSVM	C-C-LSSVM	GA-LSSVM	P-LSSVM
run = 1	9.3056	8.3541	6.8858	5.594	2.786
run = 2	8.7076	11.343	5.4844	4.8867	4.6361
run = 3	7.733	9.5406	7.592	6.1524	4.416
run = 4	8.6805	11.293	7.2655	5.3014	3.8288
run = 5	8.7599	13.787	8.068	4.1212	3.8322
run = 6	8.6594	13.388	6.04	5.5753	4.0734
run = 7	8.8136	10.493	7.9462	4.7008	4.944
run = 8	8.721	11.287	8.0008	5.74	4.4109
run = 9	7.9913	11.746	8.9826	5.9559	4.159
run = 10	7.549	10.485	7.7004	5.2602	3.417
Max.	9.3056	13.787	8.9826	6.1524	4.944
Min.	7.549	8.3541	5.4844	4.1212	2.786
Ave.	8.4291	11.171	7.3966	5.3288	4.0503
Var.	0.3017	2.6299	1.0599	0.3806	0.3905

**Table 11:** Five models predicted error based on MAPE for FM broadcasting band.

Model	M-C-LSSVM	M-F-LSSVM	C-C-LSSVM	GA-LSSVM	P-LSSVM
run = 1	0.0061	0.0295	0.0038	0.0034	0.0016
run = 2	0.0085	0.0135	0.0037	0.0038	0.0025
run = 3	0.0067	0.0088	0.0045	0.0033	0.0016
run = 4	0.0097	0.0228	0.0036	0.0038	0.0025
run = 5	0.0053	0.0225	0.004	0.0033	0.002
run = 6	0.0084	0.0138	0.004	0.0033	0.002
run = 7	0.0085	0.0076	0.004	0.0041	0.002
run = 8	0.006	0.009	0.0037	0.0038	0.0022
run = 9	0.0723	0.0109	0.0043	0.0037	0.0021
run = 10	0.0058	0.0115	0.0046	0.0037	0.0026
Max.	0.0723	0.0295	0.0046	0.0041	0.0026
Min.	0.0053	0.0076	0.0036	0.0033	0.0016
Ave.	0.0137	0.015	0.004	0.0036	0.0021
Var.	0.0004	5.00*E* − 05	1.00*E* − 07	8.00*E* − 08	1.00*E* − 07

**Table 12:** Five models predicted error based on RMSE for interphone band.

Model	M-C-LSSVM	M-F-LSSVM	C-C-LSSVM	GA-LSSVM	P-LSSVM
run = 1	0.0058	0.0063	0.0058	0.004	0.0026
run = 2	0.006	0.0064	0.0057	0.003	0.0028
run = 3	0.0056	0.0064	0.0057	0.0038	0.0025
run = 4	0.0057	0.0058	0.0056	0.0028	0.0024
run = 5	0.006	0.0063	0.0057	0.0029	0.0027
run = 6	0.0057	0.006	0.0057	0.0043	0.0023
run = 7	0.0057	0.0064	0.0057	0.0042	0.0022
run = 8	0.0057	0.0064	0.0056	0.0042	0.0027
run = 9	0.0058	0.0058	0.0057	0.005	0.0021
run = 10	0.0052	0.0064	0.0057	0.0037	0.0023
Max.	0.006	0.0064	0.0058	0.005	0.0028
Min.	0.0052	0.0058	0.0056	0.0038	0.0021
Ave.	0.0057	0.0062	0.0057	0.0038	0.0025
Var.	5.00*E* − 08	6.00*E* − 08	3.00*E* − 09	5.00*E* − 08	6.00*E* − 08

**Table 13:** Five models predicted error based on NMSE for interphone band.

Model	M-C-LSSVM	M-F-LSSVM	C-C-LSSVM	GA-LSSVM	P-LSSVM
run = 1	3.0704	3.6665	3.0476	2.5014	1.1987
run = 2	3.2591	3.713	2.9508	1.6634	1.8601
run = 3	2.8179	3.7018	2.9877	2.1748	1.5472
run = 4	2.93	3.0356	2.917	1.5306	1.5779
run = 5	3.2554	3.605	3.004	1.6102	1.2004
run = 6	2.9786	3.2563	2.9549	2.8918	1.7923
run = 7	2.93	3.694	2.9264	2.7878	1.7732
run = 8	2.9325	3.7573	2.9167	2.7969	1.0517
run = 9	3.0542	3.0584	2.9812	3.852	1.0245
run = 10	2.4479	3.7791	2.9221	2.654	1.8802
Max.	3.2591	3.7791	3.0476	3.852	1.8801
Min.	2.4479	3.0356	2.9167	1.5306	1.0245
Ave.	2.9676	3.5267	2.9608	2.4463	1.4906
Var.	0.0536	0.0855	0.0019	0.5205	0.1166

**Table 14:** Five models predicted error based on MASE for interphone band.

Model	M-C-LSSVM	M-F-LSSVM	C-C-LSSVM	GA-LSSVM	P-LSSVM
run = 1	0.0209	0.015	0.0039	0.0026	0.0015
run = 2	0.0085	0.015	0.0037	0.0032	0.0012
run = 3	0.0243	0.0118	0.0038	0.0031	0.0012
run = 4	0.0225	0.0104	0.0034	0.0027	0.0021
run = 5	0.0112	0.0132	0.0033	0.0029	0.0015
run = 6	0.0039	0.0093	0.0031	0.0031	0.0022
run = 7	0.0059	0.0123	0.0034	0.0033	0.0026
run = 8	0.009	0.0132	0.0031	0.003	0.0013
run = 9	0.0125	0.0099	0.0037	0.0036	0.023
run = 10	0.005	0.0146	0.0038	0.0031	0.0026
Max.	0.0243	0.015	0.0039	0.0036	0.0026
Min.	0.0039	0.0093	0.0031	0.0026	0.0012
Ave.	0.0124	0.0125	0.0035	0.0031	0.0019
Var.	6.00*E* − 05	4.00*E* − 06	9.00*E* − 08	8.00*E* − 08	3.00*E* − 07
